# Comparing self-report and parental report of psychopathologies in adolescents with substance use disorders

**DOI:** 10.1007/s00787-021-01865-9

**Published:** 2021-09-04

**Authors:** Sören Kuitunen-Paul, Anna Eichler, Melina Wiedmann, Lukas A. Basedow, Veit Roessner, Yulia Golub

**Affiliations:** 1 Department of Child and Adolescent Psychiatry, Faculty of Medicine, Technische Universität Dresden, Fetscherstr. 74, 01307 Dresden, Germany; 2 Department of Child and Adolescent Mental Health, Friedrich‐Alexander‐Universität Erlangen‐Nürnberg (FAU), University Hospital Erlangen, Erlangen, Germany

**Keywords:** Addiction, Behavioral problems, Emotional problems, Inter-rater agreement, Questionnaire, Substance use disorder

## Abstract

Both internalizing and externalizing psychopathologies interfere with the treatment of substance use disorders (SUD) in adolescents. Self-reports of psychopathologies are likely biased and may be validated with parental reports. We compared *N* = 70 standardized self-reports of adolescents entering outpatient SUD treatment (13.2–18.6 years old, 43% female) to parental reports on the same psychopathologies, and explored biases due to gender, age, SUD diagnoses and SUD severity. Bivariate bootstrapped Pearson correlation coefficients revealed several small to moderate correlations between both reporting sources (*r* = 0.29–0.49, all *p*_corrected_ ≤ 0.039). A repeated measures MANOVA revealed moderately stronger parental reports of adolescent psychopathologies compared to adolescent self-reports for most externalizing problems (dissocial and aggressive behaviors, *p* ≤ 0.016, *η*^2^_part_ = 0.09–0.12) and social/attention problems (*p* ≤ 0.012, *η*^2^_part_ = 0.10), but no differences for most internalizing problems (*p* ≥ 0.073, *η*^2^_part_ = 0.02–0.05). Differences were not associated with other patient or parental characteristics including age, gender, number of co-occurring diagnoses or presence/absence of a certain SUD (all *p*_uncorrected_ ≥ 0.088). We concluded that treatment-seeking German adolescents with SUD present with a multitude of extensive psychopathologies. The relevant deviation between self- and parental reports indicate that the combination of both reports might help to counteract dissimulation and other reporting biases. The generalizability of results to inpatients, psychiatry patients in general, or adolescents without SUD, as well as the validity of self- and parental reports in comparison to clinical judgements remain unknown.

**Supplementary Information:**

The online version contains supplementary material available at 10.1007/s00787-021-01865-9.

## Introduction

Adolescence is a period of experimentation, associated with increased use of psychoactive substances both legal and illegal [1]. In some cases, risky consumption patterns lead to substance-specific mental disorders, i.e. substance use disorders (SUDs) characterized by a strong desire to use the substance and the neglect of detrimental consequences on personal and social functioning. Epidemiological research has shown that SUDs typical onset is in adolescence or early adulthood [1, 2] with risky consumption patterns appearing in the months and years before disorder onset is reported [1, 3].

SUDs in adolescents are associated with diverse health-related harms including comorbid mental disorders as well as psychopathologies [4–6]. Psychopathologies include emotional and behavioral problems such as anxiety or deviant behavior [7, 8], and can be divided into externalizing and internalizing pathologies. They represent distinct obstacles for SUD therapy [9–11] that might need clinical attention already during SUD treatment [12–14]. Externalizing problems such as aggressive behavior during social interactions might be shown in intervention settings, too, thereby complicating therapeutic relationships and increasing the risk for unsuccessful treatments [15]. Internalizing behavior such as depressive behaviors might limit the adolescent’s ability to cognitively process SUD therapy contents or may manifest as inactivity during therapy sessions. Such depressive symptoms may be associated with limited therapy success [16]. The majority of adolescent SUD patients either reports clinically relevant psychopathologies or qualify for co-occurring diagnoses of mental disorders, including conduct disorders (50–74%) and depressive disorders (14–50%) [17–20]. A reliable and valid assessment of co-occurring psychopathologies is thus crucial. As of now, assessment procedures generally include self-reports by the adolescent either during interview [21] or in a questionnaire [22]. Interviews may result in increased reluctance and subsequently reduced willingness to disclose information on current behavioral or emotional problems. Questionnaires may be subject to the same biases while holding several advantages. For example, respondents may not need to fear social disapproval when revealing personal information given that no interviewer is present [23]. Unfortunately, patient self-reports may still be biased by several factors including motivation for dissimulation and sensitivity of the topic assessed [22, 23]. One option to validate patient self-reports is to compare them to parental reports for the very same psychopathologies in their children.

Earlier research has shown that, despite using the same questions and answer options for both patients and parents, their reports still deviate from each other to a certain degree. For example, associations for externalizing and internalizing behavior varied between *r* = 0.29 and *r* = 0.57 when 189 US American outpatients aged 11–17 and their parents were assessed [24]. There, parents reported stronger psychopathologies for their children than the children themselves. In a representative sample of 1757 healthy German adolescents aged 11–18 years, both reports by standardized instruments were moderately associated with adolescents reporting more problems than parents [25]. Similar findings were reported for 580 Finnish 15–16 year old general hospital outpatients and their parents, where associations were moderate to low while parents reported fewer problems for their child than the child itself [26]. Most studies found lower concordance for internalizing than for externalizing behavior problems [24, 26].

It is thus assumed that parents have access to the respective emotions and behaviors of their children, either directly through observation and self-disclosure by the child, or through second-hand information by relatives, peers, school counselors, or other community members [27]. In adolescents with SUDs, however, no study has, to our knowledge, yet assessed the degree to which parental and adolescent self-reports on psychopathologies overlap. In this subgroup of adolescents, the access of parents to the behavior and emotions of their child might be even lower, compared to children with other mental disorders. One reason is the illegality of substance use, as well as possible embarrassment and legal consequences of adolescent behavior. Adolescents will thus be motivated to underreport or hide the possession and use of the substance [28] as well as any adverse consequences as long as possible. Such adverse consequences may include multiple psychopathologies such as psychotic sensations due to stimulant use [29], depressive symptoms following the acute use of cannabis [30] or stimulants such as methamphetamine [31]. Parental reports may therefore be a valuable addition or alternative to self-reports when clinicians gather valid information for the planning of SUD treatments. In clinical settings like these, the validity of self-reports has already been questioned, at least for substance use self-reports [32, 33]. Likewise, it seems possible that self-reports and parental reports concerning psychopathologies may differ, which in turn would be relevant for the clinical interpretation of these common assessment methods.

## Research aims and hypotheses

This leads to important implications for the assessment of psychopathologies in adolescents with SUDs. We thus expect that:

1. adolescents with SUD report multiple strong psychopathologies [17–20] in comparison to general population samples for which raw sum scores are reported (*M*_YSR-total_ = 0.29–0.38) [26, 34];
2. parental reports will at best be moderately associated with self-reports [25, 26];
3. parental reports will not differ strongly from adolescent reports for externalizing psychopathologies as they can be observed well or expose the family to apparent legal consequences [24, 35];
4. parental reports will differ strongly for internalizing behavior such as feelings of anxiety or depression [24, 26].
  We aim to reduce additional biases to the results by controlling for available patient or parental characteristics. Out of the set of available characteristics, we will only include those with significant associations to the difference between parental and self-reports. Due to the exploratory nature, and given that previous studies tested for additional effects due to gender, age etc. in unselected samples [21, 26, 34], we expect that:
5. none of these variables are associated with differences between parental and self-reports [7, 36].

## Methods

### Procedure

Data collection was embedded into the standard diagnostic procedures at the Outpatient Clinic for Adolescent Substance Abuse, University Hospital C. G. Carus Dresden, Germany, see study protocol NCT03444974 registered at clinicaltrials.gov. Questionnaires were handed out to patients and their legal guardians at the first consultation appointment. The criteria for mental disorders including SUDs according to ICD-10 were assessed in a personal interview by a trained clinical psychologist. Study assessments took place before any intervention started. All procedures were conducted in accordance with the Declaration of Helsinki and were approved by the Institutional Review Board/ethics committee of the University Hospital C. G. Carus Dresden (EK 66022018). Both patients and legal guardians agreed to study participation by written consent after a comprehensive verbal and written information. Patients did not receive reimbursements for participation in analyzed assessments.

### Participants

Between November 2017 and May 2021, *N* = 275 treatment-seeking adolescents had contact with the outpatient clinic. *N* = 223 patients and their legal guardians agreed to participate in the study. We excluded patients who did not return the questionnaires (*N* = 126, 56%), whose parental and self-report were not filled out within 3 months apart from each other (*N* = 6, 2%), who did not live with at least one biological or social parent (*N* = 18, 8%), or who did not live with the parent who filled out the parental questionnaire (*N* = 3, 1%). The final sample comprised *N* = 70 adolescent SUD patients who lived with the reporting parental guardian, predominantly in a single-parent household (60%, see Suppl. Table 1). Patients were 13.2–18.6 years old (*M* = 16.0, *SD* = 1.2) with 43% females. The majority of patients (70%) qualified for two or more current SUDs (*M* = 1.9, *SD* = 0.7), predominantly due to cannabis (83%), reported a relevant amount of SUD-related problems [77% above cut-off in Drug Use Disorder Identification Test (DUDIT), *N* = 62], and qualified for one or more comorbid mental disorder (77%), predominantly conduct disorder (30%) and affective disorders (24%), see Table 1.

**Table 1:** Clinical characteristics of adolescent SUD patients (*N* = 70) regarding mental disorders according to semi-structured diagnostic assessment by a clinician

	Patients *N* (%)
ICD-10 SUDs (current) due to^a,b^	
F10 Alcohol	37 (53%)
F11 Opioids	1 (1%)
F12 Cannabinoids	58 (83%)
F14 Cocaine	1 (1%)
F15 Other stimulants, including caffeine (and methamphetamine)	31 (44%)
F16 Hallucinogens	1 (1%)
F18 Volatile solvents	1 (1%)
F19 Multiple drug use	2 (3%)
No. of SUDs according to ICD-10^a,b^	
* M*, *SD*	1.9 (0.7)
0 SUD	− (−%)
1 SUD	21 (30%)
2 SUDs	33 (47%)
3 SUDs	15 (22%)
4 SUDs	1 (1%)
Severity of SUD (Drug Use Disorder Identification Test, DUDIT sum score)	(*n* = 62)
* M*, *SD*	14.9 (9.4)
Above cut-off for severe SUD problems (i.e., 8.5 or more points)	48 (77%)
Comorbidities: Current ICD-10 mental disorders other than SUD^c,d^	
None	16 (23%)
Any of the following	54 (77%)
F00-F09 Mental disorders due to known physiological conditions (*n* = 62)	2 (3%)
F30-F39 Mood [affective] disorders	17 (24%)
F40-F49 Neurotic, stress-related and somatoform disorders	15 (21%)
F60-F69 Disorders of adult personality and behavior^e^	1 (1%)
F80-F89 Disorders of psychological development	2 (3%)
F90 Hyperkinetic disorder (i.e. attention-deficit disorder with hyperactivity)	11 (16%)
F91 Conduct disorders	21 (30%)
F92-99 Disorders starting during childhood including attention-deficit disorder without hyperactivity	11 (16%)

*SUD* Substance Use Disorder according to ICD-10, i.e., F1x.1 harmful use or F1x.2 substance dependence

^a^No patient qualified for F13 Substance use disorder due to sedatives or hypnotics

^b^F17 Nicotine use disorder was not regularly documented in included patients before December 2019, thus it is not reported here. In 2020 and 2021, all *n* = 15 patients qualified for F17 Nicotine use disorder. Likewise, the mean number of SUDs would have been higher if F17 could have been included

^c^The number of patients with valid diagnostic information is presented in brackets whenever it differed from *n* = 70. In these instances, percentages relate to this number of patients with valid information. For the calculation of the “any comorbid diagnosis” variable, cases with missing information were assumed to have no diagnosis in this field, resulting in *N* = 70 for “any comorbid diagnosis”

^d^No diagnoses from any of the following ICD-10 mental disorder classes were present: F20-F29 Schizophrenia, schizotypal and delusional disorders (*n* = 64); F50-F59 Behavioral syndromes associated with physiological disturbances and physical factors; F70-F79 Mental retardation

^e^Personality disorders may, in certain cases, be present during late adolescence (i.e. age 16 and older) although a formal diagnosis may require information beyond a structured clinical interview with the patient [1]

Parents were 32–56 years old (*M* = 41.6, SD = 5.7, not available for *N* = 12 parents, *N* = 1 parent with invalid age), predominantly female (*N* = 63, 90%) and did predominantly live apart from the other biological parent (75% combined, see Suppl. Table 1).

### Measures

#### Psychopathologies

In the Youth Self-Report (YSR/11-18) [37] for adolescents aged 11–18 years, as well as in the corresponding parental version Child Behavior Checklist (CBCL/4-18) [38], respondents rate adolescent behavioral, emotional, social and physical problems for the previous six months. Both questionnaires comprise 120 items with three response options (not applicable = 0, sometimes = 1, frequently = 2). For individual analysis, 118 of those items should be summed up, while for the comparison of parental and self-reports, the manual authors recommend that only those items are summed up whose wording is identical for the self-report and adolescent questionnaire ([39] p.7). This leaves 101 items to be analyzed (plus item 113 that provides an open-answer format for ‘other problems’). Answers are summed up to three higher-order scales which are comprised of eight subscales: *internalizing* behavior problems (social withdrawal, 7 items; somatic complaints, 9 items; anxious/depressed, 14 items), *externalizing* behavior problems (delinquent behavior, 12 items; aggressive behavior, 20 items), and *total* behavior problems (comprising all aforementioned as well as social problems, 8 items; schizoid/obsessive problems, 6 items; attention problems, 10 items; and 18 items referring to ‘other problems’ that do not constitute a sub-scale). Notably, 4 of those items are part of more than one sub-scale (items 1, 62, 45, 103). The German versions are reported to have sufficient psychometric qualities [37, 38]. Due to the focus on the 101-items-analysis, norm values (*T*-values) serving as cut-off for clinically relevant problems in German children and adolescents [35] were not calculated. Parental and self-reports were filled out within *M* = 0.5 months apart from each other (*SD* = 0.6, range = 0.0–2.9), i.e., in *n* = 62 cases (88%) were both questionnaires filled out within one month or less which equals the average duration of the outpatient diagnostic phase.

#### ICD-10 diagnoses

Diagnoses were given by a clinical psychologist, psychotherapist, or medical doctor with specialization in child and adolescent psychiatry, who assessed criteria for major mental disorders according to ICD-10 in a semi-structured clinical face-to-face interview with the patients. A SUD diagnosis was assigned when criteria for either harmful use or dependence syndrome for any relevant psychoactive substance were met.

#### Substance use disorder severity

The German version of the Drug Use Disorders Identification Test (DUDIT [40]) is validated for adolescents with SUD [41]. It is an 11-item self-report instrument identifying problems related to the use of illicit substances. Items are scored on a five-point Likert scale (items 1–9) or a three-point scale (items 10–11), resulting in an overall sum score of 0–44. Previous research in adolescents with SUD showed adequate psychometric properties [41, 42] and suggested a cut-off of 8.5 + to be indicative for SUD in adolescents [41]. Internal consistency of the instrument was large for the present sample (Cronbach’s *α* = 0.84, *n* = 51 adolescents with complete item data) and in a previous work of our group (Cronbach’s *α* = 0.87, *n* = 114 adolescent SUD outpatients) [43].

#### Sociodemographic characteristics

Information on patient and parental age, gender, education, number of children in the family, and residency were either assessed verbally by clinical staff during the initial meeting in our hospital, or using a standardized generic questionnaire at the same meeting. Response options were either free text (age in years) or forced-choice options (gender: male/female) that were subsequently concluded into categories (e.g. for education).

### Statistical analysis

Analyses were conducted with IBM SPSS Statistics 27.0. Missings were deemed completely missing at random by Littles MCAR test for CBCL items [2.1% of all items were missing, *χ*^2^ (3995) = 1778.0, *p* = 1.000] and for YSR items [2.1% of all items were missing, *χ*^2^ (3289) = 2303.5, *p* = 1.000]. Hence, YSR and CBCL missings were replaced by the respective scale mean. DUDIT missings were not completely at random for the total sample of *n* = 62 adolescents (after excluding *n* = 8 with complete missings), *χ*^2^ (88) = 118.8, *p* = 0.016. In accordance with [44], prerequisites for a single item imputation based on scale means are nonetheless fulfilled, i.e., Cronbach’s *α* > 0.70 and unidimensional items (see Ref. [41] for a dimensionality discussion). Thus the same replacement procedure was applied for the *n* = 9 adolescents with one missing item and the *n* = 2 adolescents with two missing items, effectively replacing 15 missing values out of 682 analyzed values (2.1%).

Bivariate Pearson correlation coefficients were calculated to test for significant associations between self-report and parental report in each of the three YSR/CBCL higher-order scales and eight subscales. Due to the non-normal distribution of several YSR/CBCL variables (internalizing behavior problems with all subscales, social problems, schizoid/obsessive behavior) according to the Kolmogorov–Smirnov test with Lilliefors-correction (*p* ≤ 0.006), correlation coefficients were bootstrapped (BCa-method, *N* = 1000 repetitions). To avoid alpha error inflation caused by multiple testing, *p* values were corrected after Bonferroni-Holm [45].

To test whether and which scales yielded different values comparing parental reports to self-reports, we ran a repeated measure multivariate analyses of variance (rmMANOVA) with one within-subject factor (rater: parent via CBCL vs. adolescent via YSR). To identify possible between-subject factors, we checked all other variable for significant univariate Pearson correlations with the CBCL-YSR-difference for each YSR/CBCL scale, correcting for multiple testing after Bonferroni-Holm [45]. We defined a-priori that only those variables would be used in the next step that resulted in moderate to large correlations and that significance level would not be considered given the small sample size, see Ref. [46]).

Effect sizes were classified according to Cohen [47] into small effects (|*r*|≥ 0.10, *η*_part_^2^ ≥ 0.01), moderate effects (|*r*|≥ 0.30, *η*_part_^2^ ≥ 0.06), and large effects (|*r*|≥ 0.50, *η*_part_^2^ ≥ 0.14). Increased chances for type I errors in the rmMANOVAs [48] are taken into account when interpreting the results of our non-normally distributed variables.

## Results

### Descriptive extent of problem behavior

**Fig. 1:**
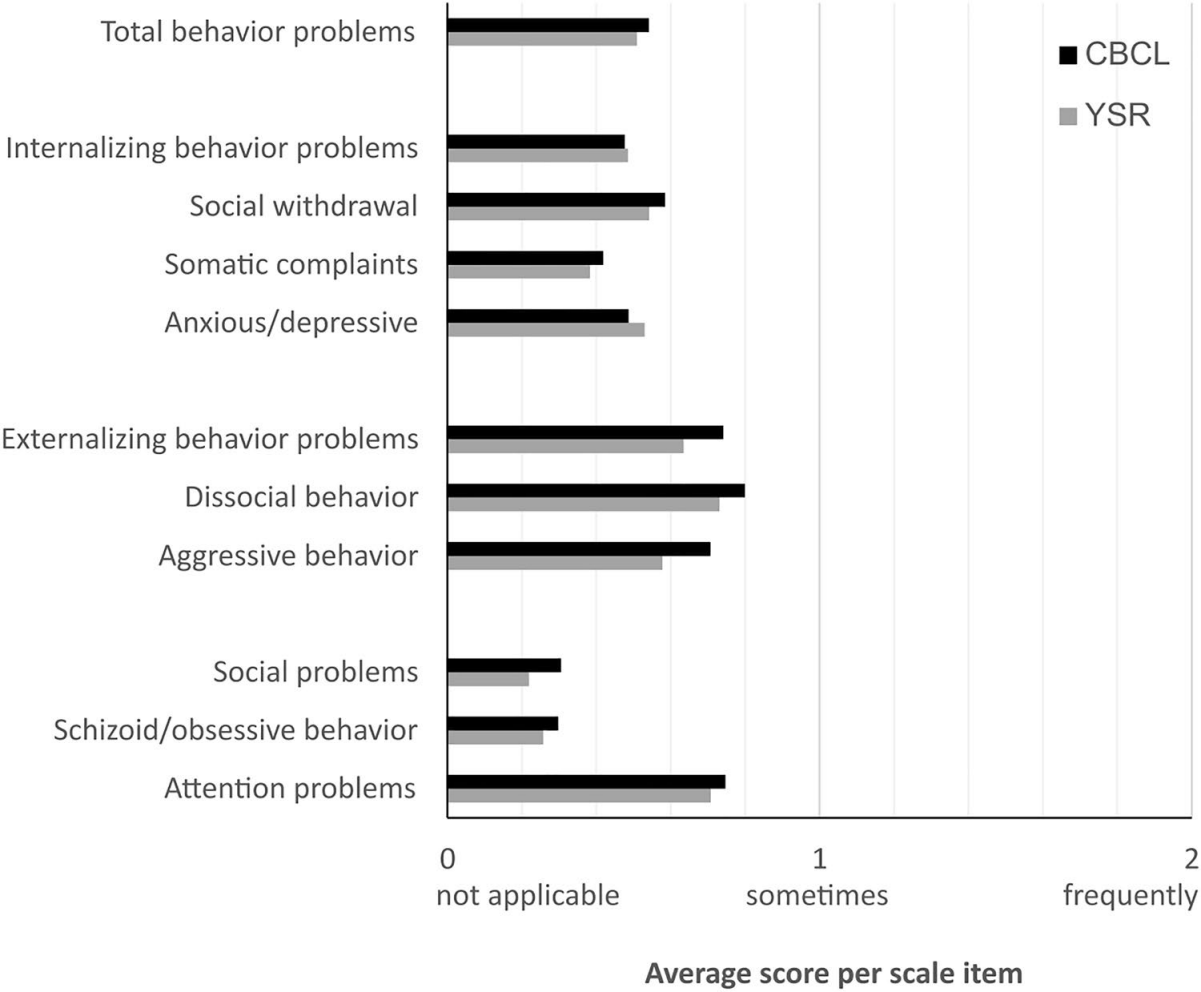
Average score per scale item, for each CBCL/YSR-scale as calculated by scale mean divided by number of items per scale. Results indicate that behavioral problems are most strongest for externalizing and attention problems. Only items included in both CBCL and YSR were included in the analysis

In line with hypothesis (1), self-reports indicated a large extent of behavioral problems (*M*_YSR-total problems_ = 51.3, see Table 2) as compared to much smaller values from general population samples (*M*_YSR-total_ = 29.8–38.6) [26, 34]. A graphical comparison between self-reports and parental reports per scale (Fig. 1) show similar patterns across sources, i.e. stronger problems especially for attention problems as well as externalizing problems including dissocial and aggressive behavior. Furthermore, Fig. 1 depicting the average score per item of a certain scale shows that reports of psychopathologies are not subject to ceiling effects.

**Table 2:** Descriptives and associations between self-reports (YSR) and parental reports (CBCL) on psychopathologies in *N* = 70 adolescent SUD patients. Only items included in both CBCL and YSR were included in the analysis

YSR/CBCL scale		Scale sum scores										Individual difference (CBCL—YSR)	
	No. of items	Parental report (CBCL)	Self-report (YSR)	Association between YSR and CBCL ^a,b^				Univariate difference (rmMANOVA with DUDIT score as covariate)					
		*M* (SD)	*M* (SD)	*r*	[95% CI]^c^	*p*_corrected_	Effect size	*F* (1,69)	*p*	η^2^_part_	Effect size	*M* (SD)	*M*_diff_*/M*_YSR_ (%)
Total behavior problems	101	54.6 (27.0)	51.3 (24.1)	0.32	[0.04, 0.56]	0.027	Moderate	6.2	0.015	0.09	Moderate	3.2 (29.7)	6%
Internalizing behavior problems	29	13.8 (8.7)	14.0 (9.9)	0.39	[0.17, 0.57]	0.005	Moderate	1.5	0.219	0.02	–	− 0.2 (10.3)	− 2%
Social withdrawal	7	4.0 (2.5)	3.8 (3.1)	0.32	[0.09, 0.51]	0.027	Moderate	8.8	0.004	0.12	Moderate	0.2 (3.3)	8%
Somatic complaints	9	3.7 (3.2)	3.4 (2.9)	0.37	[0.15, 0.56]	0.008	Moderate	3.3	0.073	0.05	–	0.3 (3.4)	9%
Anxious/depressive	14	6.8 (5.2)	7.4 (5.9)	0.45	[0.24, 0.61]	< 0.001	Moderate	1.2	0.274	0.02	–	− 0.6 (5.8)	− 8%
Externalizing behavior problems	32	23.7 (12.0)	20.3 (9.4)	0.45	[0.24, 0.62]	< 0.001	Moderate	0.2	0.624	0.004	–	3.4 (11.4)	17%
Dissocial behavior	12	9.5 (4.8)	8.7 (3.9)	0.29	[0.06, 0.49]	0.039	Small	8.8	0.004	0.12	Moderate	0.8 (5.2)	9%
Aggressive behavior	20	14.1 (8.1)	11.5 (6.2)	0.49	[0.29, 0.65]	< 0.001	Moderate	6.1	0.016	0.09	Moderate	2.5 (7.4)	22%
Social problems	8	2.4 (2.1)	1.7 (1.9)	0.34	[0.11, 0.53]	0.019	Moderate	7.3	0.009	0.10	Moderate	0.6 (2.3)	40%
Schizoid/obsessive behavior	6	1.7 (2.1)	1.5 (1.9)	0.20	[− 0.38, 0.41]	0.192	–	1.8	0.182	0.03	–	0.2 (2.5)	16%
Attention problems	10	7.4 (3.7)	7.0 (3.5)	0.16	[− 0.07, 0.38]	0.192	–	6.7	0.012	0.10	Moderate	0.3 (4.6)	6%

Only items included in both CBCL and YSR were included in the analysis

Percentages relate to the total of *N* = 70 adolescents/parents. Effect sizes after Cohen [47], only for significant associations/differences

*CBCL* Child Behavior Checklist. *SUD* Substance use disorder diagnosis. *YSR* Youth Self Report

^a^*p < 0.05, **p < 0.01, ***p < 0.001

^b^*r* bivariate Pearson correlation coefficient with *p* values corrected after Bonferroni-Holm [45]

^c^Bootstrapped 95% confidence interval (CI) for the bivariate Pearson correlation coefficient, BCa-method, *N* = 1000 repetitions

### Identifying possible correlates of the difference between parental and self-reports

Before differences between parental and self-reports are examined in the rmMANOVA, possible covariates have to be identified. These include the presence/absence of alcohol use disorder of the adolescent (*N* = 37 with vs. *N* = 33 without diagnosis), cannabis use disorder (*N* = 58 vs. *N* = 12), stimulant use disorder (*N* = 31 vs. *N* = 39, referring to caffeine as well as other stimulants such as methamphetamine), further patient characteristics (gender, age, number of SUDs, presence of comorbid disorders other than SUDs, SUD severity assessed with the DUDIT total score), and parental characteristics (gender, age, migration status, education level, number of children in the family). In line with hypothesis (5), possible covariates showed no significant correlation with CBCL-YSR-differences (all *p*_uncorrected_ ≥ 0.081, see Suppl. Table 2) or correlations were no longer significant after correcting for multiple testing (all *p*_corrected_ ≥ 0.092), thus they were therefore not included in rmMANOVA.

### Comparing parental reports to self-reports

As expected in hypothesis (2), associations between parental reports and patient self-reports were of moderate size for most scales, with *r* = 0.32–0.49, *p*_corrected_ = 0.001–0.027, see Table 2. Scales with a lack of overlap between parental and self-reports were dissocial behavior (small association), as well as schizoid/obsessive behavior and attention problems (no significant association).

The rmMANOVA confirmed large general differences between parental and self-report, with *F*_Pillai_ (11, 59) = 4.32, *p* < 0.001, *η*^2^_part_ = 0.44. Parental reports were descriptively higher, e.g. for total problems, see Table 2. In contrast to hypothesis (3), parents reported moderately stronger dissocial behavior and aggressive behavior compared to adolescent self-reports. In contrast to hypothesis (4), parental reports for internalizing behavior problems did not significantly differ from adolescent self-reports except for moderately stronger parental reports regarding social withdrawal. In addition, parents also reported moderately stronger attention problems and social problems.

## Discussion

This study analyzed reports of psychopathology, comparing self-reports by treatment-seeking adolescent outpatients with SUDs to reports by their parent. Both parents and -as expected- adolescents reported comparably strong psychopathologies especially regarding externalizing behaviors, but also attentional problems. Similarly, scale means are much higher when compared to general population studies [26, 34], e.g. with 17.0–38.6 for total problems [26] as compared to 51.3–54.6 in our sample. Reports by both sources were moderately interrelated. For specific scales, interrelations were small or negligible, thus relevant differences between parental and self-reports were found. Interestingly, parents reported significantly more behavioral problems in the same areas where adolescents themselves see the strongest problems, i.e. dissocial and aggressive behaviors as well as attention problems.

In a general population study [35], parental reports were comparable to adolescent self-reports for all internalizing problems, attention problems, and social problems, while parents reported less external behaviors, schizoid/obsessive behavior, and total behavior problems in that study. Other studies found that parents reported equal or fewer pathologies [26, 34]. In adolescents with SUD, however, we have found that psychopathologies did not go unnoticed by their parents. Instead, parents frequently reported these problems even to a larger extent (except for a non-significant difference regarding anxious/depressive problems). German adolescent outpatients with SUD may be a sample whose externalizing problems are more obvious to parents. At the same time, concordance rates were lower in our sample (correlation coefficients of 0.16–0.49) compared to concordance rates in a general population sample using the same analysis (0.36–0.68, [26]). Given the ambivalent or low motivation of adolescents to enter SUD treatment [49, 50], we suspect that adolescents in our study might have underreported the extent of current psychopathologies [22]. This is in accordance with previous results showing that adolescents dissimulate substance use in self-reports as compared to objective measures like urine testing while parental reports deviate significantly from objective reports [32, 33]. One reason for adolescent underreporting might be to present a healthier self when trying to avoid treatment or abstinence from substance use. Furthermore, this implies that the ‘actual’ rate of psychopathologies is even higher than what is reported by adolescents, and that parents might actually not overstate psychopathologies.

Contrary to hypothesis 3, parents reported higher ratings of specific external problems compared to adolescent self-reports. It has to be taken into account that adolescent SUD psychiatry patients frequently present with a history of legal problems that may either relate to substance use [8] or to the prevalent diagnosis of conduct disorder [13]. We suspect that in our sample, such common illegal actions were less frequently disclosed by adolescents who might be afraid that legal consequences might arise [23]. Parents, on the other hand, could be inclined to disclose illicit actions by their children to convince them to seek treatment for their substance use albeit they are not motivated to do so [50]. In that way, our findings might not be generalizable to samples where adolescent substance use and related problems are less obvious to parents, e.g., in samples of regular somatic care or parental counseling institutions. Anyhow, externalizing problems as well as attention and social problems are by far the dominant psychopathology in our sample, underlining a need to take those problems seriously into consideration for treatment and to frequently screen for additional mental disorders including conduct disorder and attention-deficit disorders [13].

We found no evidence that internalizing behavior in general is less likely to be reported by parents of adolescents with SUD. Correlation coefficients between self-reports and parents’ reports for subscales were lower regarding all subscales with 0.16–0.49 compared to 0.27–0.55 in Finnish adolescents [26], and regarding internal and external behavior problems with 0.39–0.45 in our study compared 0.49–0.58 in U.S.-American outpatients without SUD [24].

## Limitations

Comparability to other studies is compromised by the gender ration amongst parents. While some studies did not report parental gender at all [26, 35], others relied predominantly on fathers making up for 89.7% [51] and 100% [24] of paternal reports. There might be important differences in the mechanisms and effects of parental supervision as exhibited by fathers compared to mothers with adolescent children [52]. One study showed father-child relationship qualities were differentially related to pathology report concordance as compared to mother–child relationship qualities [53]. Furthermore, fathers may rely on second-hand information rather than first-hand disclosures from the adolescent [27]. Notably, fathers are more likely to be frequently absent from family activities, or to have left the family earlier [54]. Thus, they might have a lower likelihood of gaining reliable knowledge about psychopathologies of their child while being supposedly less likely to showing up for clinical appointments as well.

The non-normality of our outcome variables may have increased the rate of false findings (type I error), see Oberfeld, Franke [48]. However, most differences found between parental and self-reports were far below the significance threshold of *α* = 0.05, e.g. total behavior problems with *p* = 0.015. We thus assume that our results may rather represent true differences in ratings.

Unfortunately, we could not actively control for adolescent underreporting. As outlined above, both parental reports and self-reports might be biased, though differently. For example, reports of deviant behavior might be more valid or reliable if they are gathered from parents [27, 28], especially if they exhibit active methods of supervision, i.e. “direct attempts to find out about or participate in the child’s day” including parent asking the child or spouses, and parent being involved in the activity [27].

The concordance between YSR and CBCL reports may be associated with several factors that were not controlled for in this study. Such alternative explanations for the imminent differences between parental and adolescent reports include parental depressivity and anxiousness, as well as aspects of the parent–child interaction. For example, earlier studies in non-SUD settings found mothers’ levels of depressivity or anxiousness related to higher reports of child depressivity and anxiousness [34, 55]. In pre-adolescent children aged 10–12, relationship variables such as high frequency and intensity of discussions between mother and child were related to lower concordance between psychopathology reports [53]. Furthermore, while socio-economic status was associated to report concordance [34], other sociodemographics of parents and children were not, including parental education level [21] as well as age and gender of the adolescent [56].

Notably, not in all cases did parents and adolescents fill out the questionnaire simultaneously. While the mean difference of 0.5 months on this sample is still within the range of our average duration of the outpatient diagnostic phase, we cannot rule out that any kind of intervention effect might be already present within this time. As a consequence, the diagnostic process as well as any contact with therapeutic staff members may have an influence on psychopathologies or the reports of them.

The comparably small number of parent-patient couples may further limit the heterogeneity of the sample as well as decrease the a-posteriori test power, i.e., the possibility to find significant effects of additional analysis factors. This is important given that all analyzed factors, i.e. specific SUDs, were non-significant but yielded considerable effect sizes as did self-reported SUD severity. It is therefore possible that replication studies with larger samples would find that these factors would mediate the association between parental and self-reports. Furthermore, comparable studies in more diverse clinical or mixed samples [24, 26] as well as non-clinical samples [25, 35, 51] analyzed over one-hundred, sometimes several thousand parent-patient couples.

## Implications for future research

Interesting options to supplement self-reports and parental reports are to include reports from teachers, or clinical experts. Teachers could use the TRF Teacher Report Form [57] which is similar to the CBCL and YSR. On the other hand, teachers may have even more limited insight into these adolescents given that school absenteeism is a common problem among those adolescents [58] and even a symptom of SUD in the way that they “neglected major roles to use” [59]. For clinicians, some psychopathologies such as deviant behavior might be harder to judge by clinicians with limited access to everyday activities of the adolescent as compared to the parents. Nevertheless, clinical judgment is still a gold standard in the field.

## Relevance and implications

As far as we know, this is the first time that adolescent SUD outpatients and their parents were examined for the convergence and divergence in psychopathology reports. The concordance between our and earlier studies in larger clinical and non-clinical populations leads us to generalize and conclude that parental reports of psychopathology in adolescents suffering from SUD might be a sufficient alternative to adolescent self-reports. Asking parents only is a time-saving way to assess these important aspects that may help to plan SUD treatment. In the light of continuing evidence for underreporting by adolescents, we suggest interpreting parental reports as the lower bound for actual psychopathologies. It is, of course, helpful to seek additional self-reports whenever possible. The moderate to large correlations have shown that both sources report a common core of problems, while there seem to be problematic behaviors that are not consistently reported by either one of the sources. Relying on self-reports only might not reveal all aspects of psychopathologies. We thus recommend combining parental and self-reports if possible to counteract dissimulation and other reporting biases, while additionally exploring areas on relevant discordance using structured interviews or expert observations. On the other hand, adding additional information sources and assessments is always accompanied by increased demand for resources as well as an increasing need for interpretation guidelines whenever significant discordances appear. This might be even more challenging in settings with already limited resources or lacking motivation of patients and parents to collaborate. Finally, the validity of the reports as well as the generalizability of our results to inpatients, psychiatry patients in general, or adolescents without SUD remains unknown.

## Supplementary Information

Below is the link to the electronic supplementary material.

Supplementary file1 (DOCX 71 kb)

## Data Availability

Original data is part of the ongoing study. Any publication of raw data has to be permitted by the funding agency.

## Code availability

Analysis codes for rmMANOVAs and correlation analyses are available from the contact author upon request.
