# Supplementary material for: Comparing self-report and parental report of psychopathologies in adolescents with substance use disorders
Source: Eur Child Adolesc Psychiatry. 2021 Sep 4;32(2):331–42. doi: 10.1007/s00787-021-01865-9 (PMC9970943; doi:10.1007/s00787-021-01865-9)
Supplement: Supplementary file 1 — Supplementary file1 (DOCX 71 kb) [file 787_2021_1865_MOESM1_ESM.docx]

# **Supplementary Tables**

**Suppl. Table 1** Self-reported sociodemographic characteristics of parents (partly available for *N* = 58 of 70 parents).

| **Characteristic (number of parents with valid data)** | **Parents**  ***N* (%)** |
| --- | --- |
| **Migration status (*n* = 58)** | 3 (5%) |
|  |  |
| **Educational status (*n* = 57)** |  |
| 8 years or less | 9 (16%) |
| 9-10 years | 37 (65%) |
| 11 years or more | 11 (19%) |
|  |  |
| **Relationship status regarding the other biological parent patient (*n* = 59)** |  |
| Still in relationship | 15 (25%) |
| Not in relationship, never had | 8 (14%) |
| Not in relationship, split up | 34 (58%) |
| Not in relationship, deceased | 2 (3%) |
|  |  |
| **Number of biological children that both parents have together (*n* = 57)** |  |
| *M* (*SD*) | 2.5 (1.3) |
| Range | 1-7 |
|  |  |
| **Residency of the child (*n* = 58)** |  |
| With father (single) | 2 (3%) |
| With mother (single) | 33 (57%) |
| With mother and father | 14 (24%) |
| With mother and another person, e.g. step-father | 7 (12%) |
| Away from parents - foster family/adopted | 2 (3%) |
| Importantly, percentages relate to the respective number of parents with valid data (see respective sample size in brackets). Sociodemographic information was provided either by the mother/adoptive mother (*n* = 50, 87%), by the father (*n* = 6; 10%), or the person filling out the questionnaire was not specified (*n* = 2; 3%). | |

**Suppl. Table 2**  Correlates of differences between self-reports (YSR) and parental reports (CBCL) on psychopathologies, in *N* = 70 adolescent SUD patients. Only items included in both CBCL and YSR were included in the analysis.

| **Difference between CBCL and YSR for each scale** | **Adolescent patient‘s characteristics** | | | | | | | | | | | | | | | | | | |
| --- | --- | --- | --- | --- | --- | --- | --- | --- | --- | --- | --- | --- | --- | --- | --- | --- | --- | --- | --- |
|  | **AUD** | | | **CUD** | | | **StUD** | | | **Number  of SUDs ^a^** | | | | **SUD severity (DUDIT)** | | | **Any comorbid diagnosis** | | |
|  | ***n* = 70** | | | ***n* = 70** | | | ***n* = 70** | | | ***n* = 70** | | | | ***n* = 62** | | | ***n* = 70** | | |
|  | ***r*** | **[95% CI]^g^** | ***p*** | ***r*** | **[95% CI]^g^** | ***p*** | ***r*** | **[95% CI]^g^** | ***p*** | | ***r*** | **[95% CI]^g^** | ***p*** | ***r*** | **[95% CI]^g^** | ***p*** | ***r*** | **[95% CI]^g^** | ***p*** |
| **Total behavior problems** | .05 | [-.18; .28] | .675 | .03 | [-.19; .27] | .750 | -.14 | [-.36; .09] | .219 | | -.06 | [-.29; .17] | .582 | -.31 | [-.50; -.04] | **.007^c^** | .14 | [-.09; .36] | .237 |
|  |  |  |  |  |  |  |  |  |  | |  |  |  |  |  |  |  |  |  |
| **Internalizing behavior problems** | .15 | [-.08; .37] | .212 | .04 | [-.19; .27] | .726 | -.09 | [-.32; .14] | .418 | | .01 | [-.22; .24] | .918 | -.21 | [-.44; .03] | .087 | .10 | [-.13; .32] | .395 |
| **Social withdrawal** | .02 | [-.21; .25] | .842 | .06 | [-.17; .29] | .598 | -.10 | [-.33; .13] | .389 | | -.04 | [-.27; .19] | .716 | -.22 | [-.44; .02] | .081 | .04 | [-.19; .27] | .697 |
| **Somatic complaints** | .07 | [-.16; .30] | .552 | -.02 | [-.25; .21] | .859 | -.07 | [-.29; .16] | .567 | | -.03 | [-.26; .19] | .760 | -.12 | [-.36; .12] | .333 | .14 | [-.09; .36] | .236 |
| **Anxious/depressive** | .19 | [-.04; .40] | .107 | .07 | [-.16; .30] | .545 | -.06 | [-.29; .16] | .568 | | .07 | [-.16; .30] | .533 | -.16 | [-.40; .08] | .188 | .09 | [-.14; .32] | .421 |
|  |  |  |  |  |  |  |  |  |  | |  |  |  |  |  |  |  |  |  |
| **Externalizing behavior problems** | -.03 | [-.26; .19] | .761 | .04 | [-.19; .27] | .728 | -.19 | [-.40; .04] | .111 | | -.12 | [-.35; .11] | .288 | -.27 | [-.48; -.02] | **.034 ^d^** | .16 | [-.07; .38] | .168 |
| **Dissocial behavior** | -.04 | [-.28; .18] | .690 | -.02 | [-.25; .21] | .870 | -.18 | [-.40; .05] | .126 | | -.16 | [-.38; .07] | .166 | -.33 | [-.53; -.08] | .**008 ^e^** | .13 | [-.10; .35] | .274 |
| **Aggressive behavior** | -.02 | [-.25; .21] | .860 | .07 | [-.15; .30] | .513 | -.16 | [-.38; .07] | .177 | | -.07 | [-.30; .16] | .530 | -.18 | [-.41; .06] | .149 | .16 | [-.07; .38] | .173 |
|  |  |  |  |  |  |  |  |  |  | |  |  |  |  |  |  |  |  |  |
| **Social problems** | .05 | [-.18; .28] | .651 | -.01 | [-.24; .22] | .927 | -.03 | [-.26; .20] | .787 | | .04 | [-.19; .27] | .715 | -.20 | [-.43; .04] | .109 | .18 | [-.05; .40] | .123 |
| **Schizoid/obsessive behavior** | -.01 | [-.24; .22] | .916 | .01 | [-.21; .25] | .890 | -.10 | [-.32; .13] | .409 | | -.11 | [-.34; .12] | .342 | -.15 | [-.38; .10] | .238 | .07 | [-.16; .30] | .531 |
| **Attention problems** | -.00 | [-.23; .23] | .976 | .06 | [-.17; .29] | .602 | -.12 | [-.35; .10] | .286 | | -.08 | [-.31; .15] | .493 | -.32 | [-.53; -.08] | **.009 ^f^** | .08 | [-.15; .31] | .479 |
| Significant univariate correlations are highlighted as bold.  *AUD* Alcohol use disorder diagnosis. *CBCL* Child Behavior Checklist. *CUD* Cannabis use disorder diagnosis. *DUDIT* Drug Use Disorders Identification Test. *StUD* Stimulant use disorder diagnosis, relating to cocaine, methamphetamine, speed and other stimulants. *YSR* Youth Self Report. *p* uncorrected for multiple testing. *r* bivariate Pearson correlation coefficient.  ^a^ number of SUD diagnoses without F17.x nicotine use disorder.  ^b^ *p*-value corrected after Bonferroni-Holm [1]: *p*_corrected_ = .154.  ^c^ *p*-value corrected after Bonferroni-Holm [1]: *p*_corrected_ = .129.  ^d^ *p*-value corrected after Bonferroni-Holm [1]: *p*_corrected_ = .269.  ^e^ *p*-value corrected after Bonferroni-Holm [1]: *p*_corrected_ = .092.  ^f^ *p*-value corrected after Bonferroni-Holm [1]: *p*_corrected_ = .093.  ^g^ bootstrapped 95% confidence interval (CI) for the bivariate Pearson correlation coefficient, BCa-method, *N* = 1000 repetitions. | | | | | | | | | | | | | | | | | | | |

1. Gaetano J (2018) Holm-Bonferroni sequential correction: An Excel calculator [Microsoft Excel workbook]. . 1.3 edn.,

**Suppl. Table 2 (continued)**

| **Difference between CBCL and YSR for each scale** | **Adolescent patient‘s characteristics** | | | | | |  | **Parental characteristics** | | | | | | | | | | | | | | |
| --- | --- | --- | --- | --- | --- | --- | --- | --- | --- | --- | --- | --- | --- | --- | --- | --- | --- | --- | --- | --- | --- | --- |
|  | **Age** | | | **Female gender** | | |  | **Age** | | | **Female gender** | | | **Migration** | | | **Education level** | | | **Number of children** | | |
|  | ***n* = 70** | | | ***n* = 70** | | |  | ***n* = 57** | | | ***n* = 70** | | | ***n* = 58** | | | ***n* = 57** | | | ***n* = 57** | | |
|  | ***r*** | **[95% CI]^g^** | ***p*** | ***r*** | **[95% CI]^g^** | ***p*** |  | ***r*** | **[95% CI]^g^** | ***p*** | ***r*** | **[95% CI]^g^** | ***p*** | ***r*** | **[95% CI]^g^** | ***p*** | ***r*** | **[95% CI]^g^** | ***p*** | ***r*** | **[95% CI]^g^** | ***p*** |
| **Total behavior problems** | .08 | [-.15; .30] | .504 | -.16 | [-.38; .07] | .164 |  | -.04 | [-.30; .21] | .724 | .03 | [-.20; .26] | .781 | -.01 | [-.26; .24] | .938 | -.01 | [-.27; .25] | .934 | .00 | [-.25; .26] | .949 |
|  |  |  |  |  |  |  |  |  |  |  |  |  |  |  |  |  |  |  |  |  |  |  |
| **Internalizing behavior problems** | .03 | [-.20; .26] | .776 | -.16 | [-.38; .07] | .178 |  | -.13 | [-.38; .13] | .319 | .05 | [-.18; .28] | .646 | -.10 | [-.35; .16] | .445 | -.01 | [-.26; .26] | .938 | .04 | [-.21; .30] | .731 |
| **Social withdrawal** | -.01 | [-.24; .22] | .936 | -.14 | [-.36; .09] | .227 |  | .00 | [-.25; .26] | .976 | -.02 | [-.25; .21] | .865 | -.08 | [-.33; .18] | .540 | .00 | [-.33; .18] | .999 | .08 | [-.17; .33] | .516 |
| **Somatic complaints** | .09 | [-.14; .31] | .454 | -.16 | [-.38; .07] | .168 |  | -.05 | [-.31; .20] | .688 | .08 | [-.15; .30] | .513 | -.00 | [-.26; .25] | .972 | -.08 | [-.14; .36] | .533 | .07 | [-.19; .32] | .593 |
| **Anxious/depressive** | .01 | [-.21; .25] | .880 | -.11 | [-.33; .12] | .350 |  | -.20 | [-.43; .06] | .128 | .05 | [-.18; .28] | .654 | -.14 | [-.38; .11] | .272 | .11 | [-.30; .21] | .388 | -.03 | [-.29; .22] | .801 |
|  |  |  |  |  |  |  |  |  |  |  |  |  |  |  |  |  |  |  |  |  |  |  |
| **Externalizing behavior problems** | .14 | [-.09; .36] | .234 | -.05 | [-.28; .18] | .658 |  | .02 | [-.23; .28] | .837 | .02 | [-.21; .25] | .839 | .00 | [-.25; .26] | .988 | -.04 | [-.21; .30] | .736 | -.06 | [-.31; .20] | .644 |
| **Dissocial behavior** | .09 | [-.13; .32] | .415 | .09 | [-.14; .32] | .437 |  | -.17 | [-.41; .09] | .193 | .00 | [-.23; .23] | .981 | -.07 | [-.32; .18] | .588 | .04 | [-.33; .18] | .743 | .07 | [-.18; .33] | .559 |
| **Aggressive behavior** | .15 | [-.08; .37] | .215 | -.15 | [-.37; .08] | .216 |  | .16 | [-.10; .40] | .217 | .03 | [-.20; .26] | .794 | .05 | [-.20; .30] | .693 | -.08 | [-.14; .36] | .545 | -.14 | [-.39; .12] | .278 |
|  |  |  |  |  |  |  |  |  |  |  |  |  |  |  |  |  |  |  |  |  |  |  |
| **Social problems** | -.13 | [-.35; .10] | .271 | -.12 | [-.35; .11] | .292 |  | -.01 | [-.27; .24] | .923 | -.10 | [-.33; .13] | .392 | .05 | [-.20; .31] | .659 | .12 | [-.36; .14] | .366 | .10 | [-.16; .35] | .437 |
| **Schizoid/obsessive behavior** | .05 | [-.18; .28] | .653 | -.14 | [-.36; .09] | .249 |  | -.16 | [-.40; .10] | .232 | .20 | [-.03; .41] | .088 | .08 | [-.17; .33] | .520 | -.11 | [-.41; .09] | .388 | .03 | [-.23; .28] | .814 |
| **Attention problems** | .10 | [-.13; .32] | .405 | **-.29** | [-.49; -.05] | **.014 ^b^** |  | .09 | [-.17; .34] | .488 | -.10 | [-.33; .13] | .392 | .18 | [-.07; .42] | .161 | -.17 | [-.16; .35] | .204 | -.01 | [-.27; .24] | .890 |
| Significant univariate correlations are highlighted as bold.  *AUD* Alcohol use disorder diagnosis. *CBCL* Child Behavior Checklist. *CUD* Cannabis use disorder diagnosis. *DUDIT* Drug Use Disorders Identification Test. *StUD* Stimulant use disorder diagnosis, relating to cocaine, methamphetamine, speed and other stimulants. *YSR* Youth Self Report. *p* uncorrected for multiple testing. *r* bivariate Pearson correlation coefficient.  ^a^ number of SUD diagnoses without F17.x nicotine use disorder.  ^b^ *p*-value corrected after Bonferroni-Holm [1]: *p*_corrected_ = .154.  ^c^ *p*-value corrected after Bonferroni-Holm [1]: *p*_corrected_ = .129.  ^d^ *p*-value corrected after Bonferroni-Holm [1]: *p*_corrected_ = .269.  ^e^ *p*-value corrected after Bonferroni-Holm [1]: *p*_corrected_ = .092.  ^f^ *p*-value corrected after Bonferroni-Holm [1]: *p*_corrected_ = .093.  ^g^ bootstrapped 95% confidence interval (CI) for the bivariate Pearson correlation coefficient, BCa-method, *N* = 1000 repetitions. | | | | | | | | | | | | | | | | | | | | | | |
